# Psychometric properties of the Chinese version of scales of knowledge, attitude, and practice of self-care for patients with arteriovenous fistula: a translation and verification study

**DOI:** 10.3389/fpubh.2025.1588271

**Published:** 2025-04-28

**Authors:** Chuang Li, Youbei Lin, Jiaxin Cheng, Danfeng Xu, Lan Zhang

**Affiliations:** ^1^Department of Nursing, First Affiliated Hospital of Jinzhou Medical University, Jinzhou, Liaoning, China; ^2^School of Nursing, Jinzhou Medical University, Jinzhou, Liaoning, China

**Keywords:** arteriovenous fistula, dialysis, end stage renal disease, psychometric assessment, health literacy and knowledge

## Abstract

**Background:**

Chronic kidney disease (CKD) represents a significant global public health challenge, characterized by its high prevalence and the complexity of its treatment, which collectively impose substantial burdens on patients’ quality of life and healthcare systems. Hemodialysis remains a critical life-sustaining treatment for CKD patients, with arteriovenous fistulas (AVFs) being the most commonly used vascular access. The long-term functionality of AVFs relies heavily on patients’ self-care abilities, encompassing their knowledge of maintenance practices, appropriate attitudes toward care, and the implementation of effective self-care behaviors. Consequently, accurately assessing the self-care capabilities of patients with AVFs is essential for optimizing treatment outcomes and enhancing their overall quality of life.

**Objective:**

This study aims to translate the scales of knowledge, attitude, and practice of self-care for patients with arteriovenous fistula (SKAPS-AVF) into Chinese and evaluate its psychometric properties among Chinese patients to ensure its validity and reliability in clinical and research settings.

**Method:**

The study employed the Brislin translation model to translate and back-translate the original scale, followed by cultural adaptation to ensure its relevance to the Chinese context. Exploratory factor analysis (EFA) and confirmatory factor analysis (CFA) were conducted to assess the structural validity of the scale. Reliability was evaluated by calculating Cronbach’s alpha, split-half reliability, and McDonald’s Omega (*Ω*) to determine the internal consistency and stability of the scale.

**Results:**

Exploratory factor analysis (EFA) revealed that the translated scale has a three-factor structure, with eigenvalues greater than 1 for all factors and a total variance explanation rate of 63.099%. Confirmatory factor analysis (CFA) demonstrated good model fit, with fit indices such as the chi-square value, Comparative Fit Index (CFI), Tucker–Lewis Index (TLI), and Root Mean Square Error of Approximation (RMSEA) meeting acceptable standards. Reliability analysis indicated that Cronbach’s alpha, split-half reliability, and McDonald’s Omega values all exceeded 0.7, suggesting good internal consistency and stability of the scale.

**Conclusion:**

The C-SKAPS-AVF demonstrated good psychometric properties, with high structural validity and reliability, making it a robust tool for assessing self-care capabilities in patients with arteriovenous fistulas. This scale provides a reliable measurement tool for related clinical interventions and research. However, future studies should consider expanding the sample size and evaluating the scale’s longitudinal stability and applicability across different cultural contexts.

## 1 Introduction

Chronic kidney disease (CKD) refers to structural or functional abnormalities of the kidneys caused by various factors, including glomerulosclerosis, diabetic nephropathy, and nephrotic syndrome (1). Currently, CKD affects 8–16% of the global population and is recognized as a significant public health issue worldwide (2). According to World Health Organization statistics, the global prevalence of CKD is increasing annually and is projected to become the seventh leading cause of death globally by 2030 (3). The prevalence of CKD in China is approximately 13%, making it a major public health concern. It has emerged as a significant threat to human health, alongside cancer, diabetes, and cardiovascular diseases (4). In the advanced stages of CKD, particularly end-stage renal disease (ESRD), patients typically require treatment through hemodialysis or kidney transplantation (5). In regions with limited healthcare resources, hemodialysis is the most commonly utilized treatment due to its relatively high accessibility (6). Hemodialysis involves vascular surgery to create a blood flow pathway by connecting a superficial artery and vein, allowing the patient’s blood to be filtered through a dialyzer to remove waste, fluids, and electrolytes, thereby compensating for kidney function (7). However, AVFs are frequently associated with complications such as infection, thrombosis, stenosis, and dysfunction (8). These issues not only compromise dialysis efficacy but also significantly diminish patients’ quality of life and increase healthcare expenditures. Studies have shown that many of these complications are closely linked to patients’ insufficient knowledge and inadequate self-care practices (9, 10), including failure to monitor fistula status, improper use of the affected limb, and poor hygiene maintenance. Therefore, enhancing patients’ self-management abilities, particularly by improving their understanding of AVFs care and their ability to apply this knowledge in daily practice, is essential for extending the functional lifespan of the fistula and effectively preventing related complications.

Arteriovenous fistulas are widely regarded as the optimal vascular access for hemodialysis patients, with their functional status directly impacting the adequacy of dialysis treatment (11). Studies have shown that the effectiveness and longevity of AVFs heavily depend on the patient’s ability to perform proper self-care (12). Alarmingly, 90% of dialysis patients exhibit insufficient self-care behaviors, and only 33.3% respond appropriately to AVF complications (13). These findings highlight the critical need for hemodialysis patients to establish and develop effective AVF self-care practices to maintain the optimal function of their fistulas and ensure the efficacy of dialysis therapy.

At present, the existing evaluation tools are still insufficient to evaluate the level of self-care ability of arteriovenous fistula in hemodialysis patients. The self-management questionnaire for hemodialysis patients compiled by Curtin in 2004 is not suitable for China patients due to the differences in cultural background and medical system (14). In addition, the self-management behavior rating scale for hemodialysis patients designed by Yu (15) only contains one item about the protection behavior of internal fistula, and the content is relatively simple. Similarly, the self-management scale of hemodialysis patients developed by Song and Lin (16) mainly focuses on the overall self-management ability of patients, and the evaluation of internal fistula nursing behavior is not comprehensive enough. To sum up, although the existing tools cover many aspects of self-management of hemodialysis patients, the specific evaluation of arteriovenous fistula nursing is still insufficient.

In 2023, Brazilian scholar Pessoa developed the Scales of Knowledge, Attitude, and Practice of Self-Care for patients with arteriovenous fistula (SKAPS-AVF). This scale is comprehensive, targeted, and practical, assessing patients’ self-care abilities across three dimensions: knowledge, attitude, and practice. It provides a crucial basis for personalized care and interventions (17). Therefore, this study aims to translate the SKAPS-AVF into Chinese and validate its psychometric properties among Chinese patients with arteriovenous fistulas, addressing the limitations of existing tools while providing a theoretical reference for the care of these patients.

## 2 Methods

### 2.1 Study design and participants

The Strengthening the Reporting of Observational Studies in Epidemiology (STROBE) checklist for reporting observational studies was followed in this study (18), which used a cross-sectional design. Using convenience sampling, patients from the hemodialysis unit of the First Affiliated Hospital of Jinzhou Medical University in Liaoning Province, China, were recruited between December 2024 and March 2025. Following Kendall’s recommendation that the sample size for psychometric evaluation should be 5–10 times the number of scale items (19), and accounting for a 10% attrition rate, the target sample size for the 31-item scale was set at a minimum of 341 participants. Additionally, according to guidelines, a minimum sample size of 100 is required for exploratory factor analysis (EFA), and at least 200 for confirmatory factor analysis (CFA) (20). Therefore, this study plans to recruit 350 participants as the sample. This study distributed two rounds of questionnaires: the first round took place from September to October 2024, with 150 questionnaires distributed (Sample 1). The second round was conducted from November 2024 to January 2025, with 200 questionnaires distributed (Sample 2).

The inclusion criteria for participants were: (1) age ≥18 years; (2) maintenance hemodialysis (MHD) patients using AVF as their dialysis access; and (3) undergoing regular hemodialysis for more than 3 months. Exclusion criteria: (1) severe comorbid conditions such as malignant tumors; and (2) inability to cooperate due to mental illness or low adherence.

### 2.2 Introduction of research tools

#### 2.2.1 SKAPS-AVF

This scale was developed by Professor Pessoa and his research team in 2023 (17). It consists of 31 items divided into three subscales: Knowledge: Includes two dimensions—development needs (items 2 ~ 8, 11, 13) and health deviation requirements (items 1, 9, 10, 12, 14 ~ 19). Attitude: a single-dimension subscale (items 20 ~ 23). Practice: includes two dimensions—development needs (items 24 ~ 26, which are reverse-scored items) and health deviation requirements (items 27 ~ 31). Each item is rated using a 5-point Likert scale, with a total score ranging from 31 to 155. A higher score indicates a higher level of self-care ability in patients. The scale’s explained variance and McDonald’s omega values are as follows: Knowledge (19 items): 40.4%/0.896, Attitude (4 items): 60.7%/0.843, Practice (8 items): 36.9%/0.702.

#### 2.2.2 The general information questionnaire

The general information questionnaire is designed by the researcher through reading a large number of documents, including gender, age, ethnic group, marital status, working status, education level, medical insurance, residence and Fistula location. These variables were selected based on their potential impact on self-care behaviors in hemodialysis patients.

#### 2.2.3 Chinese version of scales of knowledge, attitude, and practice of self-care for patients with arteriovenous fistula (C-SKAPS-AVF)

C-SKAPS-AVF contains 31 items, which are divided into three dimensions: Self-Care Knowledge (items 1 ~ 19), Self-Care attitude (items 20 ~ 23) and Self-Care practice (items 24 ~ 31, of which 24, 25 and 26 are reverse scoring questions); each item uses Likert’s 5-level scoring method, with the total score ranging from 31 to 155. The higher the score, the higher the patient’s self-care ability.

### 2.3 Translation and cross-cultural adaptation

**Figure 1:**
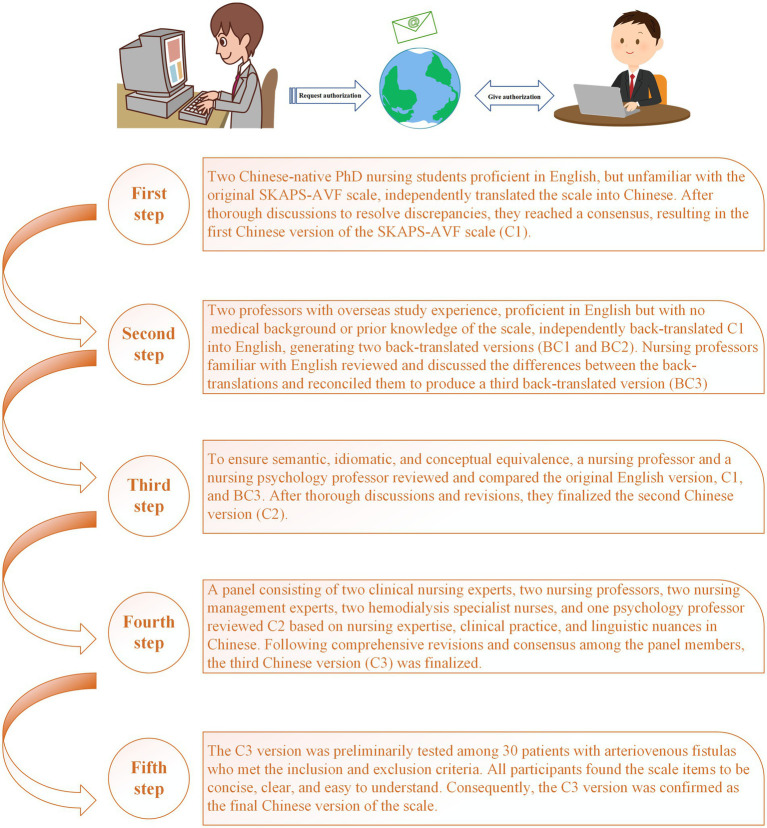
Flowchart of translation and cross-cultural adaptation of SKAPS-AVF.

Permission to translate the SKAPS-AVF scale was obtained from the original author via email. Following this, the scale was translated using a forward-backward translation process based on Brislin’s guidelines (21). The steps are as shown in Figure 1.

### 2.4 Data collection

After obtaining approval from the hospital’ relevant management departments, two specialized nurses from the hemodialysis unit were selected and trained as investigators for this study. Eligible participants meeting the inclusion and exclusion criteria were identified, and the study’s purpose was explained to them using standardized instructions, emphasizing voluntary participation and confidentiality. According to relevant studies (22, 23), given that patients are prone to anxiety mood before and after hemodialysis sessions, the survey was conducted 30 min after each hemodialysis session to ensure participants were in an optimal mental state for questionnaire completion. For patients with impaired vision or those who were older adult and frail, the investigators asked each question aloud based on the questionnaire and assisted with completing the responses. If participants had any questions during the process, the investigators provided immediate clarification to ensure full understanding. The completed questionnaires were collected immediately after being filled out.

### 2.5 Data analysis

Both AMOS 26.0 and SPSS 27.0 were used for analysis of the data. The sociodemographic traits and scale scores were derived using descriptive statistics. The mean (M) and standard deviation (SD) were used to represent variables with a normal distribution, and the median (M) and interquartile range (P25, P75) were used to report data that was not normally distributed. Frequency and percentage (%) were used to denote categorical variables. When the skewness and kurtosis scores were between −1 and +1, the data were deemed to be regularly distributed (24). With the goal to assess the psychometric qualities of the C-SKAPS-AVF, this study performed item, validity, and reliability analyses.

#### 2.5.1 Items analysis

The total scores of the C-SKAPS-AVF were ranked in descending order and divided into high-score and low-score groups, representing the top 27% and bottom 27% of scores, respectively. An independent samples t-test was used to compare the differences in each item between the high and low groups. A critical ratio (CR) > 3.000 (25) indicated that the item had high discriminative power. Item-total correlations and Cronbach’s alpha if an item was deleted were analyzed to evaluate whether each item of the C-SKAPS-AVF should be retained. An item-total correlation threshold of 0.3 (26) was used as the criterion for item inclusion.

#### 2.5.2 Reliability analysis

Cronbach’s alpha, McDonald’s Omega, and split-half reliability were used to assess the reliability of the scale. A value of ≥0.70 for Cronbach’s alpha, McDonald’s Omega, and split-half reliability was considered an acceptable standard for reliability coefficients (27). Split-half reliability was evaluated by dividing the items of the scale into two halves based on odd-even item order and calculating the correlation between the two halves.

#### 2.5.3 Validity analysis

##### 2.5.3.1 Content validity

To assess each item on the translated scale from the standpoint of content relevance, nine nursing specialists were invited to participate (28). The scale content validity index (S-CVI) and item content validity index (I-CVI) were calculated using the Delphi method. Each item in the C-SKAPS-AVF was evaluated for relevance using a 4-point Likert scale, with 1 defining no relevance and 4 defining great relevance. The percentage of experts who gave an item a rating of three or four out of all the experts was known as the I-CVI. The average of all I-CVIs across the scale was used to compute the S-CVI. High content validity was defined as I-CVI > 0.780 and S-CVI > 0.900 (29).

##### 2.5.3.2 Structure validity

The validity of the C-SKAPS-AVF was examined using EFA and CFA. To check whether EFA and CFA were appropriate for the data, Samples 1 and 2 were subjected to the Kaiser–Meyer–Olkin (KMO) test and Bartlett’s test of sphericity. Sample 1 was used to conduct an EFA to analyze the factor structure of the scale. First, we performed a promax rotation and found that the correlations between factors were all below 0.3 (30). According to relevant studies (31, 32), principal component analysis and varimax rotation are commonly used in EFA due to their ability to reduce cross-loadings and produce a clearer factor structure. Therefore, we adopted this method while examining eigenvalues and scree plot results. A KMO score > 0.60 and a Bartlett’s test result of *p* < 0.05 were considered suitability criterion (33). Principal component analysis (PCA) was used to extract factors with eigenvalues greater than 1, and items with factor loadings less than 0.50 were eliminated (34). CFA was conducted on the remaining 200 participants (Sample 2) in order to check model fit and validate the proposed factor model using maximum likelihood estimation. The following standards were taken into consideration: CMIN/DF < 3, root mean square error of approximation (RMSEA) < 0.05, root mean square residual (RMR) < 0.05, and goodness-of-fit indices including GFI, adjusted GFI (AGFI), comparative fit index (CFI), normed fit index (NFI), incremental fit index (IFI), and Tucker–Lewis index (TLI) > 0.90. Furthermore, tests of discriminant and convergent validity were conducted to assess the items’ structural validity (35). Standards: Good convergent validity is indicated by an average variance extracted (AVE) value > 0.5 and a composite reliability (CR) value > 0.7. The Fornell–Larcker criterion states that a scale has sufficient discriminant validity when the square root of the AVE for a construct is higher than its correlation coefficients with other constructs (36).

## 3 Results

### 3.1 Basic characteristics of study participants

A total of 350 participants were included in the study, with a mean age of 57.37 years (SD = 13.68). Males accounted for 60.9% (213) and females 39.1% (137). The sample consisted of 207 Han Chinese (59.1%) and 143 participants from non-Han ethnic groups (40.9%). Married individuals made up 84% (294). Among the participants, 64% (224) were employed, and 36% (126) were retired. The highest education level for most participants was a college degree or above (61.1%), and 88.6% had medical insurance coverage. Rural residents accounted for 53.1%, while urban residents constituted 46.9%. The distribution of arteriovenous fistula locations was nearly balanced, with 52.6% on the left side and 47.4% on the right. The sample showed a relatively balanced distribution regarding gender, ethnicity, and residential areas. However, there was a notable concentration in higher education attainment and medical insurance coverage, reflecting the socioeconomic characteristics of the study population (Table 1).

**Table 1:** Demographic characteristics of the population (*n* = 350).

Variables		Frequency	Percentage (%)
Gender	Males	213	60.9
	Females	137	39.1
Marriage status	Married	294	84.0
	Other	56	16.0
Ethnic group	Han	207	40.9
	National minority	143	59.1
Working state	Incumbency	224	64.0
	Retirement	126	36.0
Education level	Junior high school and below	31	8.9
	Senior high school	105	30
	College and above	214	61.1
Medical insurance	Yes	310	88.6
	No	40	11.4
Residence	Countryside	186	53.1
	Cities	164	46.9
Fistula location	Left	184	52.6
	Right	166	47.4

### 3.2 Scale translation and cross-cultural adaptation

Our team completed the scale translation following Brislin’s guidelines. During the cross-cultural adaptation process, experts noted that most items aligned well with Chinese cultural norms, with only minor adjustments needed for a few items. First, experts suggested modifying “How much do you know wash the fistula arm with soap and water immediately before starting hemodialysis?” to “How much do you know wash the fistula arm with water immediately before starting hemodialysis?” This change reflects the common practice in China, where patients are typically advised to keep the fistula area clean and dry. If cleaning is necessary, they are often recommended to use clean water rather than soap, especially harsh soaps, as soap residue might remain on the skin and affect sterile procedures during dialysis (37). Additionally, experts proposed modifying “How often do you check if the fistula arm is pale, painful or sore?” to “How often do you check if the fistula arm is pale, painful, sore or swelling?” The addition of “swelling” ensures comprehensive assessment, as swelling can indicate blood flow obstruction or infection—common symptoms requiring attention during fistula care (38). Including this term could enhance patients’ ability to identify issues early, reducing the risk of complications. The study team made the decision to follow these professional suggestions. All participants in a pilot research reported satisfaction with the scale’s semantics and scoring methodology and confirmed that it had a logical thematic framework.

### 3.3 Items analysis

First, the mean (SD) scores, as well as the skewness and kurtosis values for the 31 items of the C-SKAPS-AVF, are presented in Table 2. The dataset has a normal distribution, according to the skewness and kurtosis statistics. Second, there are significant differences between the high-score and low-score groups for each item (*p* < 0.001), with the critical ratio (CR) values for the 31 items ranging from 6.310 to 16.814. Neither 0 nor the 95% CI are included for these differences. The 95% CI excludes zero, and the correlation coefficients between the scores of each item and the overall C-SKAPS-AVF score range from 0.400 to 0.755 (*p* < 0.001). Lastly, the C-SKAPS-AVF has a Cronbach’s alpha coefficient of 0.940. Since removing any item did not cause the Cronbach’s alpha to rise over 0.940, every item was kept (Table 2).

**Table 2:** Results of normality test, reliability, critical ratio and Pearson’s correlation coefficient for C-SKAPS-AVF (*n* = 350).

Items	Mean (SD)	Skewness/Kurtosis	Correlation of amended entries to totals	Clone Bach after deletion Alpha	Cronbach’s alpha	Critical ratio (CR)	95% CI	Pearson correlation coefficient	95% CI
KN1	3.00(1.00)	−0.109/−0.258	0.676	0.937	0.968	13.615**	1.404–1.880	0.704**	0.647–0.753
KN2	3.03(1.02)	−0.003/0.282	0.732	0.937		15.925**	1.605–2.058	0.749**	0.699–0.792
KN3	3.01(0.97)	−0.470/0.079	0.680	0.937		14.444**	1.445–1.902	0.708**	0.651–0.757
KN4	3.07(1.01)	−0.058/0.025	0.693	0.937		15.120**	1.519–1.975	0.720**	0.666–0.767
KN5	3.00(1.01)	−0.143/−0.154	0.701	0.937		16.187**	1.571–2.008	0.728**	0.675–0.774
KN6	3.00(0.98)	−0.136/−0.152	0.652	0.938		13.266**	1.299–1.753	0.681**	0.621–0.734
KN7	3.01(1.01)	−0.320/0.316	0.688	0.937		13.951**	1.410–1.874	0.716**	0.660–0.763
KN8	2.96(0.99)	−0.040/0.020	0.657	0.937		13.015**	1.322–1.794	0.686**	0.626–0.738
KN9	2.97(0.96)	−0.143/−0.064	0.680	0.937		15.590**	1.489–1.921	0.707**	0.650–0.756
KN10	2.99(1.01)	0.096/0.189	0.703	0.937		14.579**	1.456–1.912	0.730**	0.677–0.775
KN11	2.96(0.988)	−0.212/0.108	0.678	0.937		14.949**	1.453–1.895	0.706**	0.649–0.755
KN12	3.01(1.03)	−0.097/0.286	0.698	0.937		15.537**	1.526–1.969	0.726**	0.672–0.772
KN13	3.07(0.95)	−0.284/−0.083	0.732	0.937		16.283**	1.508–1.924	0.755**	0.706–0.797
KN14	2.99(0.97)	−0.324/0.123	0.716	0.937		16.470**	1.520–1.933	0.741**	0.690–0.785
KN15	2.99(1.02)	−0.324/0.543	0.699	0.937		16.123**	1.552–1.985	0.726**	0.673–0.772
KN16	2.97(0.98)	−0.172/−0.110	0.694	0.937		15.136**	1.483–1.928	0.721**	0.666–0.768
KN17	2.98(0.96)	−0.031/−0.176	0.719	0.937		16.814**	1.533–1.941	0.744**	0.693–0.787
KN18	2.94(0.99)	−0.286/0.358	0.714	0.937		14.579**	1.465–1.924	0.739**	0.688–0.783
KN19	3.03(0.97)	−0.039/−0.058	0.728	0.937		16.784**	1.551–1.965	0.752**	0.703–0.794
AT20	3.05(1.07)	−0.161/0.338	0.300	0.940	0.887	6.310**	0.651–1.224	0.401**	0.227–0.441
AT21	3.03(1.04)	−0.129/−0.279	0.322	0.941		7.058**	0.721–1.279	0.400*	0.227–0.458
AT22	3.01(1.00)	−0.080/0.323	0.300	0.941		6.509**	0.624–1.166	0.411**	0.238–0.424
AT23	2.98(1.06)	−0.179/−0.097	0.302	0.941		6.462**	0.665–1.250	0.406**	0.258–0.442
PR24	3.12(1.05)	0.126/−0.013	0.400	0.940	0.936	8.168**	0.862–1.411	0.441**	0.352–0.521
PR25	3.01(1.02)	0.133/−0.142	0.379	0.940		8.139**	0.845–1.386	0.425**	0.336–0.508
PR26	3.04(0.97)	0.338/−0.061	0.356	0.940		7.622**	0.749–1.272	0.401**	0.309–0.485
PR27	3.08(1.00)	0.232/−0.230	0.395	0.940		8.029**	0.818–1.351	0.440**	0.351–0.521
PR28	3.05(1.02)	0.144/−0.411	0.397	0.940		8.434**	0.879–1.416	0.442**	0.354–0.523
PR29	3.05(1.10)	0.091/0.037	0.418	0.940		9.144**	1.040–1.612	0.466**	0.379–0.544
PR30	3.04(1.04)	0.007/0.101	0.430	0.940		8.761**	0.971–1.535	0.475**	0.389–0.552
PR31	3.05(1.07)	0.189/−0.004	0.409	0.940		8.882**	0.942–1.479	0.456**	0.368–0.535
Cronbach’s alpha	–	–	–	–	0.940	–	–	–	–
Slit-half reliability	–	–	–	–	0.700	–	–	–	–
McDonald Omega	–	–	–	–	0.930	–	–	–	–

*p < 0.05, **p < 0.01.

### 3.4 Reliability analysis

The Cronbach’s alpha coefficient for the C-SKAPS-AVF is 0.940, with reliability values for the three dimensions being 0.968, 0.887, and 0.936, respectively. Additionally, McDonald’s Omega is 0.937, and the split-half reliability is 0.700. These results indicate that the overall reliability of the C-SKAPS-AVF is good (Table 2).

### 3.5 Validity analysis

#### 3.5.1 Content validity

In this study, 9 experts were invited to evaluate C-SKAPS-AVF. The results show that I-CVI ranges from 0.880 to 1.00, and S-CVI/Ave is 0.903. C-SKAPS-AVF shows acceptable content validity.

#### 3.5.2 EFA

**Figure 2:**
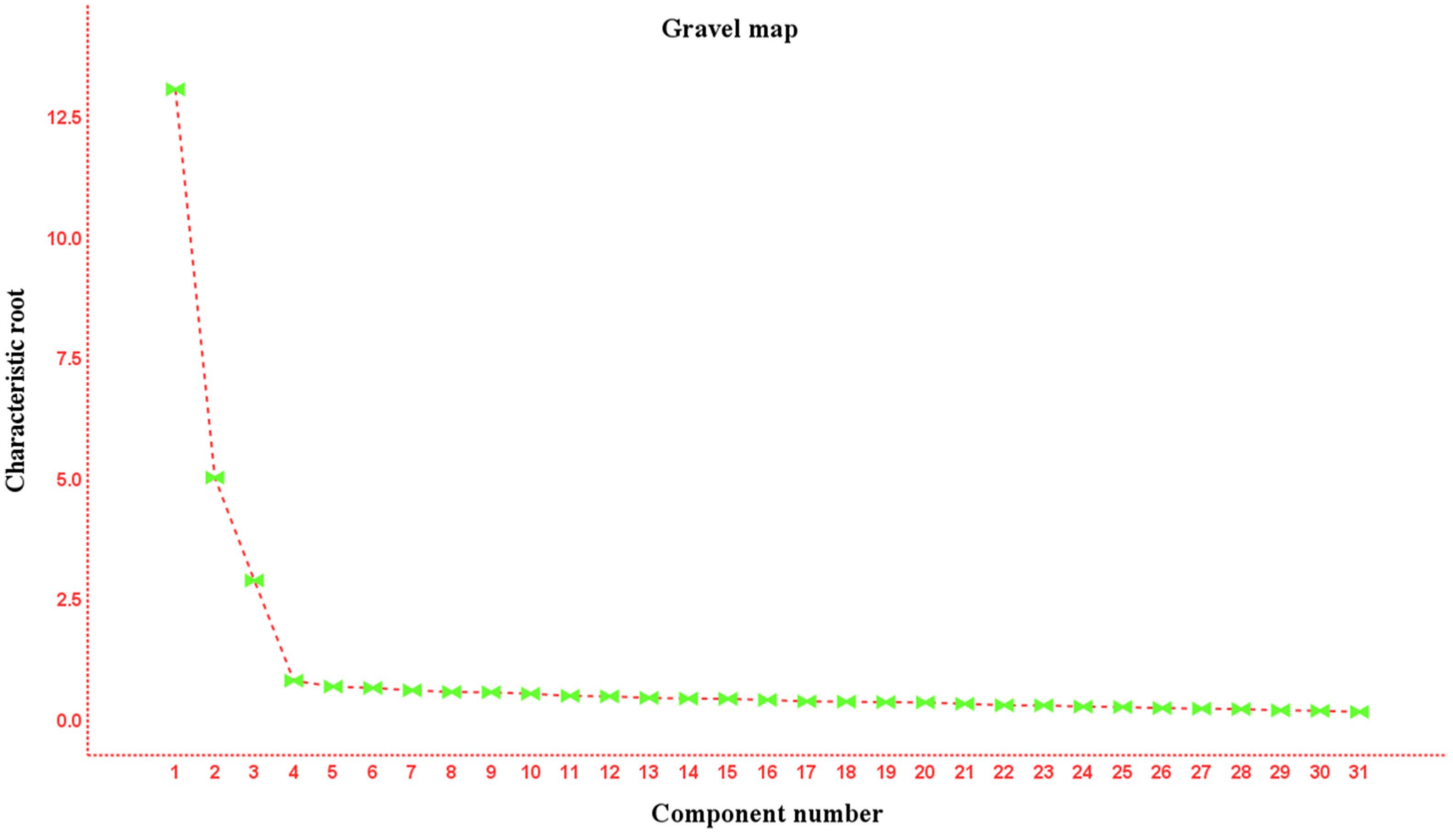
C-SKAPS-AVF’s gravel map (*n* = 150).

The results of the EFA showed that the Kaiser-Meyer-Olkin (KMO) value was 0.943, and Bartlett’ s test of sphericity was significant (*χ*^2^ = 3624.906, df = 465, *p* < 0.001). This indicates that the correlation matrix was not an identity matrix, making it suitable for factor extraction. Using principal component analysis with varimax rotation, three factors with eigenvalues greater than 1 were extracted automatically, as the decline in the scree plot became less pronounced after the third point (Figure 2). These three factors formed the dimensions of the scale: self-care knowledge (items 1 ~ 19), self-care attitudes (items 20 ~ 23), and self-care practices (items 24 ~ 31), explaining 67.290% of the total variance. The component matrix showed that all factor loadings were greater than 0.50, so no items were removed (Table 3).

**Table 3:** Factor loadings for the three dimensions of the C-SKAPS-AVF (*n* = 350).

Items	F1	F2	F3
KN1	0.784		
KN2	0.810		
KN3	0.768		
KN4	0.815		
KN5	0.758		
KN6	0.746		
KN7	0.766		
KN8	0.785		
KN9	0.766		
KN10	0.796		
KN11	0.705		
KN12	0.813		
KN13	0.761		
KN14	0.833		
KN15	0.792		
KN16	0.811		
KN17	0.803		
KN18	0.804		
KN19	0.824		
AT20		0.880	
AT21		0.838	
AT22		0.859	
AT23		0.876	
PR24			0.812
PR25			0.830
PR26			0.795
PR27			0.849
PR28			0.807
PR29			0.850
PR30			0.813
PR31			0.822

#### 3.5.3 CFA

**Figure 3:**
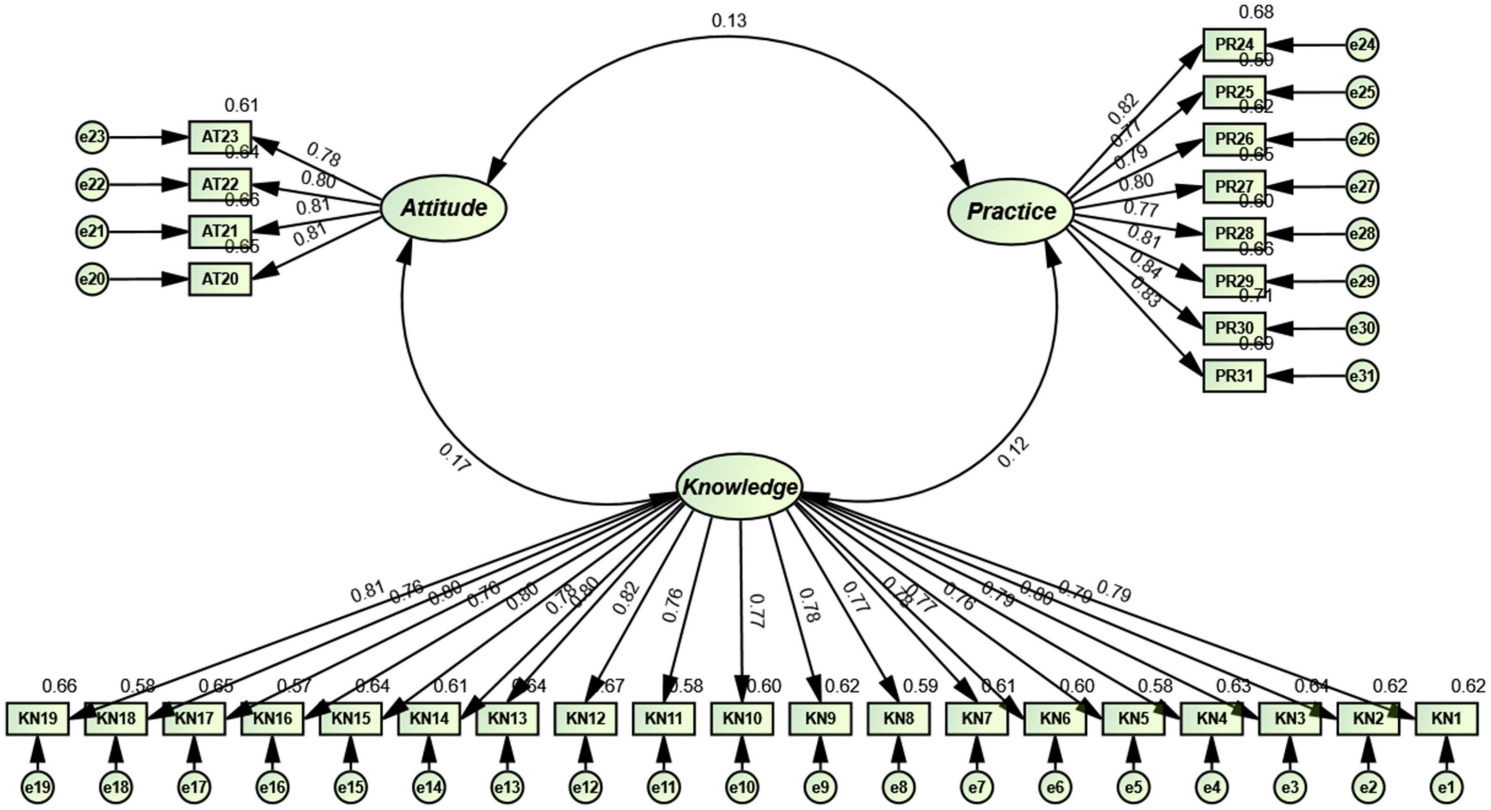
Standardized three-factor loading map for C-SKAPS-AVF (*n* = 200).

For Sample 2, the Kaiser-Meyer-Olkin (KMO) value was 0.951, and Bartlett’s test of sphericity was significant (*χ^2^* = 4654.237, df = 465, *p* < 0.001), indicating suitability for factor analysis. Confirmatory factor analysis (CFA) was performed on the Sample 2 data (*n* = 200) using the maximum likelihood method. The results demonstrated that the three-factor model of the C-SKAPS-AVF exhibited excellent fit indices: CMIN/DF = 1.067, RMSEA = 0.018, RMR = 0.037, AGFI = 0.900, NFI = 0.907, IFI = 0.994, TLI = 0.993, CFI = 0.993, and GFI = 0.900. Additionally, the composite reliability (CR) ranged between 0.878 and 0.967, and the average variance extracted (AVE) values were 0.641–0.671. The correlation between factors ranged from 0.090 to 0.649. The square roots of the AVEs are all greater than the correlation coefficients between the corresponding dimensions (Table 4). In addition, all standardized factor loading values were >0.5 (Figure 3).

**Table 4:** Convergent and discriminant validity of C-SKAPS-AVF (*n* = 350).

Convergent validity						Discriminant Validity					
Factors	Paths	Items	Std. Estimate	S.E.	*P*	AVE	CR	Factors	F1	F2	F3
F1	→	KN1	0.785		***	0.671	0.967	F1	0.617		
F1	→	KN2	0.787	0.082	***						
F1	→	KN3	0.800	0.078	***						
F1	→	KN4	0.794	0.079	***						
F1	→	KN5	0.763	0.082	***						
F1	→	KN6	0.772	0.079	***						
F1	→	KN7	0.783	0.082	***						
F1	→	KN8	0.769	0.076	***						
F1	→	KN9	0.785	0.076	***						
F1	→	KN10	0.773	0.083	***						
F1	→	KN11	0.758	0.081	***						
F1	→	KN12	0.820	0.078	***						
F1	→	KN13	0.802	0.076	***						
F1	→	KN14	0.779	0.077	***						
F1	→	KN15	0.797	0.084	***						
F1	→	KN16	0.756	0.078	***						
F1	→	KN17	0.804	0.075	***						
F1	→	KN18	0.764	0.080	***						
F1	→	KN19	0.811	0.073	***						
F2	→	AT20	0.808	0.082	***	0.641	0.878	F2	0.118	0.641	
F2	→	AT21	0.811	0.083	***						
F2	→	AT22	0.800	0.078	***						
F2	→	AT23	0.783	0.071	***						
F3	→	PR24	0.824		***	0.649	0.937	F3	0.090	0.106	0.649
F3	→	PR25	0.768	0.065	***						
F3	→	PR26	0.787	0.065	***						
F3	→	PR27	0.805	0.066	***						
F3	→	PR28	0.773	0.070	***						
F3	→	PR29	0.811	0.073	***						
F3	→	PR30	0.844	0.066	***						
F3	→	PR31	0.833	0.071	***			√AVE	0.785	0.801	0.806

***P < 0.01; √AVE, Square root of AVE.

## 4 Ethical considerations

The study was approved by the Ethics Committee of Jinzhou Medical University (JZMULL2025057). All participants signed an informed consent form before responding. In addition, all information obtained from the participants was strictly confidential and anonymous, and the data collected were used only for research purposes.

## 5 Discussion

According to relevant studies, arteriovenous fistula (AVF) serves as the lifeline for hemodialysis patients, and its functionality directly impacts the effectiveness of dialysis treatment and the quality of life for patients (39). However, due to a lack of knowledge about AVF care, negative attitudes, and improper care practices, many patients face numerous challenges in actual care, resulting in a high incidence of AVF-related complications. Existing self-care assessment tools are often general in nature. While they offer advantages in evaluating overall patient self-management abilities, they exhibit limitations when addressing the specific domain of AVF care. The SKAPS-AVF scale, developed by Pessoa et al. in 2023, comprehensively assesses patients’ self-care abilities from the dimensions of knowledge, attitude, and practice. This targeted tool provides a reliable foundation for personalized education and intervention strategies. During the localization of this scale into Chinese, we strictly adhered to Brislin’s translation principles, incorporating forward translation, back-translation, and cultural adaptation procedures to ensure that the language accurately reflected the core essence of the original scale within the Chinese cultural context. Furthermore, statistical analyses demonstrated that the C-SKAPS-AVF exhibited excellent reliability and validity, confirming its effectiveness in accurately assessing the self-care abilities of Chinese patients regarding AVF.

Firstly, compared to the commonly used Self-Care Ability Scale for Arteriovenous Fistula in Hemodialysis Patients (SAS-AVF-HDP) in China, the item design of the C-SKAPS-AVF demonstrates a higher level of detail, effectively covering critical aspects of patient self-care. For example, Item 11: “How much do you know about washing the arm with water immediately before starting hemodialysis?” assesses patients’ hygiene practices prior to dialysis. In contrast, the SAS-AVF-HDP does not explicitly address this specific care detail. By emphasizing handwashing and local hygiene, this item enhances patients’ awareness of infection prevention, aligning with the core goal of reducing infection risks in hemodialysis patients (40). Furthermore, the C-SKAPS-AVF not only emphasizes knowledge acquisition but also prioritizes the ability to translate knowledge into practice. For example, Item 18:“What measures should be taken to check whether the arteriovenous fistula is functioning properly?” explicitly guides patients to verify fistula function through specific techniques such as palpation and auscultation. Compared to the SAS-AVF-HDP, this item places greater emphasis on assessing practical skills. This design not only reflects the patient’ s ability to manage their condition but also assists healthcare providers in identifying weaknesses in the patient’ s execution of self-care tasks. Additionally, the C-SKAPS-AVF incorporates the evaluation of patients’ ability to respond to emergencies, further enhancing its comprehensiveness. Item 19: “How much do you know about managing an arteriovenous fistula when no thrill/vibration is detected?” focuses directly on handling arteriovenous fistula dysfunction, highlighting patients’ emergency response capabilities (41). In contrast, the SAS-AVF-HDP primarily emphasizes general behaviors, such as maintaining cleanliness and avoiding external forces, lacking specific descriptions of emergency scenarios. Therefore, C-SKAPS-AVF is more effective in guiding patients to address critical issues like fistula dysfunction. Lastly, the C-SKAPS-AVF is structured according to the dimensions of “knowledge,” “attitude,” and “practice,” showcasing a logical and systematic approach. For example, item 13: “How much do you know about how long it takes for an arteriovenous fistula to stop bleeding after needle removal?” clearly focuses on knowledge assessment, helping patients understand the importance of hemostasis through specific time-related guidance. This complements the attitude and practice sections of the scale. In contrast, the SAS-AVF-HDP has less defined item classifications, and certain items (e.g., those related to complication prevention) may lead to overlap or confusion. Similarly, item 17: “How much do you know about the presence of blood clots in the arteriovenous fistula?” directly prompts patients to monitor for blood clot formation, a high-risk issue. This approach encourages patients to take an active role in identifying potential complications (42). On the other hand, the SAS-AVF-HDP primarily emphasizes healthcare provider-led guidance, with less focus on patients’ ability to independently recognize and address disease risks. The advantage of the C-SKAPS-AVF lies in its more detailed item design, more scientific categorization, and comprehensive coverage of patients’ abilities from knowledge and attitude to practice. It is particularly superior in areas such as handling emergencies and proactive self-management. This scale is better suited for comprehensive nursing assessments of arteriovenous fistula patients, enabling more effective guidance for personalized nursing interventions and improving patients’ nursing abilities and quality of life.

Firstly, this study found that the variance explained by the original scale’s knowledge and practice subscales was 40.4 and 36.9%, respectively, both below 50% (43). This indicates that the explanatory power of its factor structure was relatively limited, and the two-factor structure of these subscales was not well supported. This may suggest that the original scale had low measurement validity in the knowledge and practice dimensions, possibly due to cross-loading between items or instability in dimensional classification. However, after localization and cultural adaptation, improved language clarity likely enhanced Chinese patients with arteriovenous fistulas’ understanding of the items, reducing measurement error. Additionally, cultural adaptation increased the relevance of the items to this patient population, strengthening their correlation with the underlying factors. As a result, although the self-care knowledge and self-care practice subscales exhibited a single-factor structure different from the original, their cumulative variance contribution rates increased to 63.629 and 69.058%, respectively, with all factor loadings exceeding 0.7 (44). The total variance explained by the 31-item scale reached 67.290%, significantly higher than that of the original scale. These findings suggest that the adapted scale demonstrates stronger explanatory power and structural stability in assessing self-care knowledge, attitudes, and practices. Overall, its structural validity has improved, enabling a more accurate evaluation of patients’ self-care knowledge and practice abilities. This highlights the importance of appropriate revisions and adaptations in cross-cultural scale applications, as such modifications can enhance the reliability and validity of measurement tools, making them more suitable for evaluating self-care in Chinese patients with arteriovenous fistulas.

Secondly, item analysis is a crucial step in evaluating the quality of each item within a scale, aiming to determine whether the items are appropriate and independent within a dimension or scale (45). By comparing the results between high and low groups for each item, the C-SKAPS-AVF demonstrated significant discriminatory ability in assessing participants’ self-care knowledge, attitudes, and practices. Furthermore, the item-total correlations for all items exceeded the minimum standard threshold, indicating that these items are representative and contribute meaningfully to the overall scale. Validity refers to the degree to which an instrument accurately corresponds to the real-world phenomena it aims to measure (46). We assessed the scale’s validity through analyses of content and construct validity. The Delphi method revealed that both the I-CVI and S-CVI exceeded reference thresholds, indicating that the scale adequately and accurately reflects the levels of self-care knowledge, attitudes, and practices among AVF patients. Additionally, robust construct validity was demonstrated through EFA and CFA. The EFA results revealed a three-factor structure, with all factors having eigenvalues greater than 1, and a total variance explained rate of 63.099% (47). This indicates that the localized scale effectively captures the primary dimensions of the target construct, with an ideal level of variance explained. Moreover, all item factor loadings exceeded 0.4, demonstrating strong associations between individual items and their respective factors, further validating the scale’s ability to measure the intended dimensions. In summary, C-SKAPS-AVF exhibited strong construct validity from both theoretical and statistical perspectives, making it a reliable tool for self-care research in AVF patients, with promising application prospects. The CFA results further confirmed the structural validity of the localized C-SKAPS-AVF. All fit indices fell within acceptable ranges, indicating a well-fitting model that accurately reflects the expected factor structure. Specifically, indicators such as, CFI, TLI and RMSEA met standard thresholds, verifying the scale’s structural validity. Furthermore, the results of convergent and discriminant validity analyses provided additional support for the scale’s effectiveness. Convergent validity indicated a high level of consistency among items within each factor, demonstrating the scale’s capability to reliably measure the distinct dimensions of self-care knowledge, attitudes, and practices. Discriminant validity confirmed sufficient differentiation between the dimensions, minimizing overlap among factors and enhancing the scale’s ability to distinguish between different aspects of self-care. This is particularly crucial in AVF patient care research, where clear differentiation among knowledge, attitudes, and practices is essential for providing precise clinical intervention tools.

Finally, in this study, the reliability of the C-SKAPS-AVF was comprehensively examined. Reliability refers to the consistency and stability of measurement results (48), indicating whether repeated measurements under the same conditions yield consistent outcomes. To assess the reliability of the C-SKAPS-AVF, internal consistency testing was conducted for the overall scale and its three dimensions. The results showed that Cronbach’s alpha values were all greater than 0.7, indicating a high level of internal consistency for the scale and its subdimensions.

Moreover, split-half reliability testing and McDonald’s Omega values also exceeded 0.7, further validating the reliability of the scale. These findings demonstrate that the scale can stably and accurately measure the levels of self-care knowledge, attitudes, and practices among arteriovenous fistula patients, exhibiting excellent repeatability and consistency.

## 6 Conclusion

The findings from multiple analyses indicate that the C-SKAPS-AVF demonstrates high reliability and validity in measuring self-care knowledge, attitudes, and practices among patients with arteriovenous fistulas. This tool effectively captures patients’ knowledge levels, attitude changes, and behavioral practices during the self-care process. The introduction and application of the C-SKAPS-AVF not only fill a gap in the evaluation tools available in this field in China but also provide clinical nurses and administrators with a scientific and comprehensive assessment instrument. Through this tool, weak points in patients’ knowledge, attitudinal biases, and issues in care practices can be identified, enabling healthcare providers to develop more targeted health education programs.

## 7 Limitations

This study has several limitations. First, the sample was drawn from a specific region, which may limit its representativeness and affect the generalizability of the scale. Second, while the study validated the reliability of the scale, it did not assess the stability of longitudinal measurements or the scale’s sensitivity in real-world interventions, which may restrict its applicability for dynamic monitoring. Additionally, this study used Kaiser’ s criterion (eigenvalue > 1) to determine the number of factors in the EFA but did not employ parallel analysis to further verify the objectivity of factor retention. Although parallel analysis is widely regarded as a more robust method for determining factor structure (49), its omission in this study may introduce some subjectivity in factor extraction. However, previous research on scale adaptation has demonstrated that a two-stage approach combining EFA and CFA effectively evaluates the psychometric properties of cross-cultural scales (31, 50). After establishing the factor structure through EFA, this study further confirmed model fit using CFA, with results meeting psychometric criteria, supporting the validity of the methodological approach. Future research should incorporate more objective factor retention methods, such as parallel analysis, to enhance the methodological rigor of cross-cultural scale validation. Additionally, further studies should test the stability of the factor structure in larger or more diverse samples to strengthen its generalizability. Finally, we acknowledge that the use of convenience sampling may introduce selection bias and limit the generalizability of findings to broader hemodialysis populations. While this approach enhanced feasibility given resource constraints, future studies employing probability sampling methods would strengthen external validity.

## Data Availability

The raw data supporting the conclusions of this article will be made available by the authors, without undue reservation.
